# Supplementary material for: UHPLC-Q-Orbitrap-MS-Based Metabolomics Reveals Chemical Variations of Two Types of Rhizomes of Polygonatum sibiricum
Source: Molecules. 2022 Jul 22;27(15):4685. doi: 10.3390/molecules27154685 (PMC9331047; doi:10.3390/molecules27154685)
Supplement: Supplementary file 1 [file molecules-27-04685-s001.zip › Metabolic profile of PS_03_SI.pdf]

Table S1. Basic information of the two types of plants

	Wide type (Wtype)	Evergreen type (Gtype)
Weight per plant (g)	42.0±21.9	329.3±90.0
High per plant (mm)	623.3±133.3	786.7±75.1
Thickness of stem (mm)	6.3±1.1	7.0±1.0
Aboveground characteristics	Single pole independent growth, withering in winter	Multi-rod clump growth, growing in all seasons
Tiller	One new tiller sprout per rhizome each year, rarely more than one	
Underground rhizome growth trend	Horizontal, growing in one direction	Horizontal, growing in one direction in the shape of ginger
Number of rhizomes	1	Same as the number of stems on the ground
Yield per 4 years	68.6 g/plant	1861.7 g/plant
Polysaccharide content	9.33%	7.91%

Figure S1.

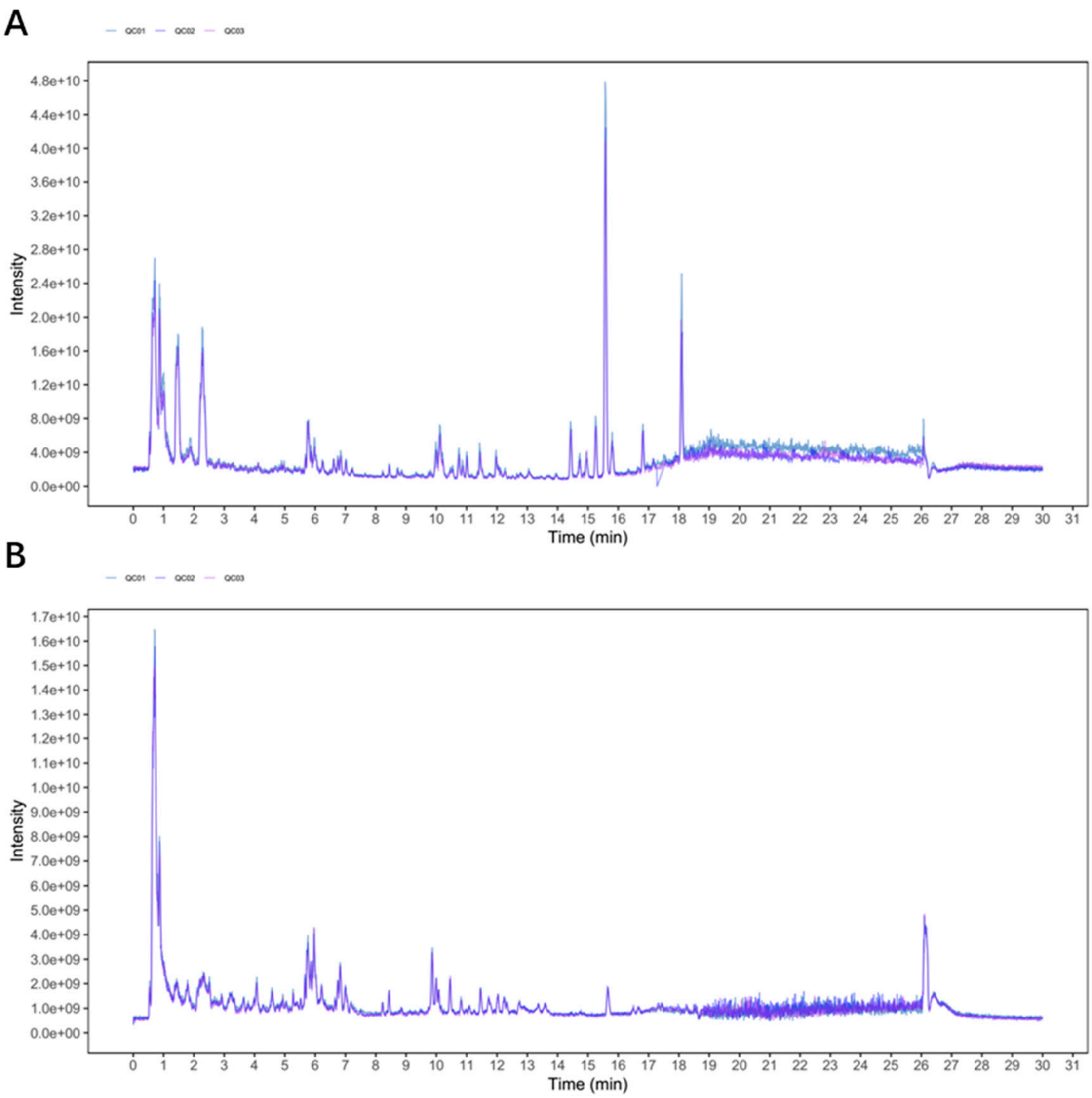

Figure S1. Base peak chromatograms of QC sample in positive (A) and negative (B) ionization modes

Figure S2

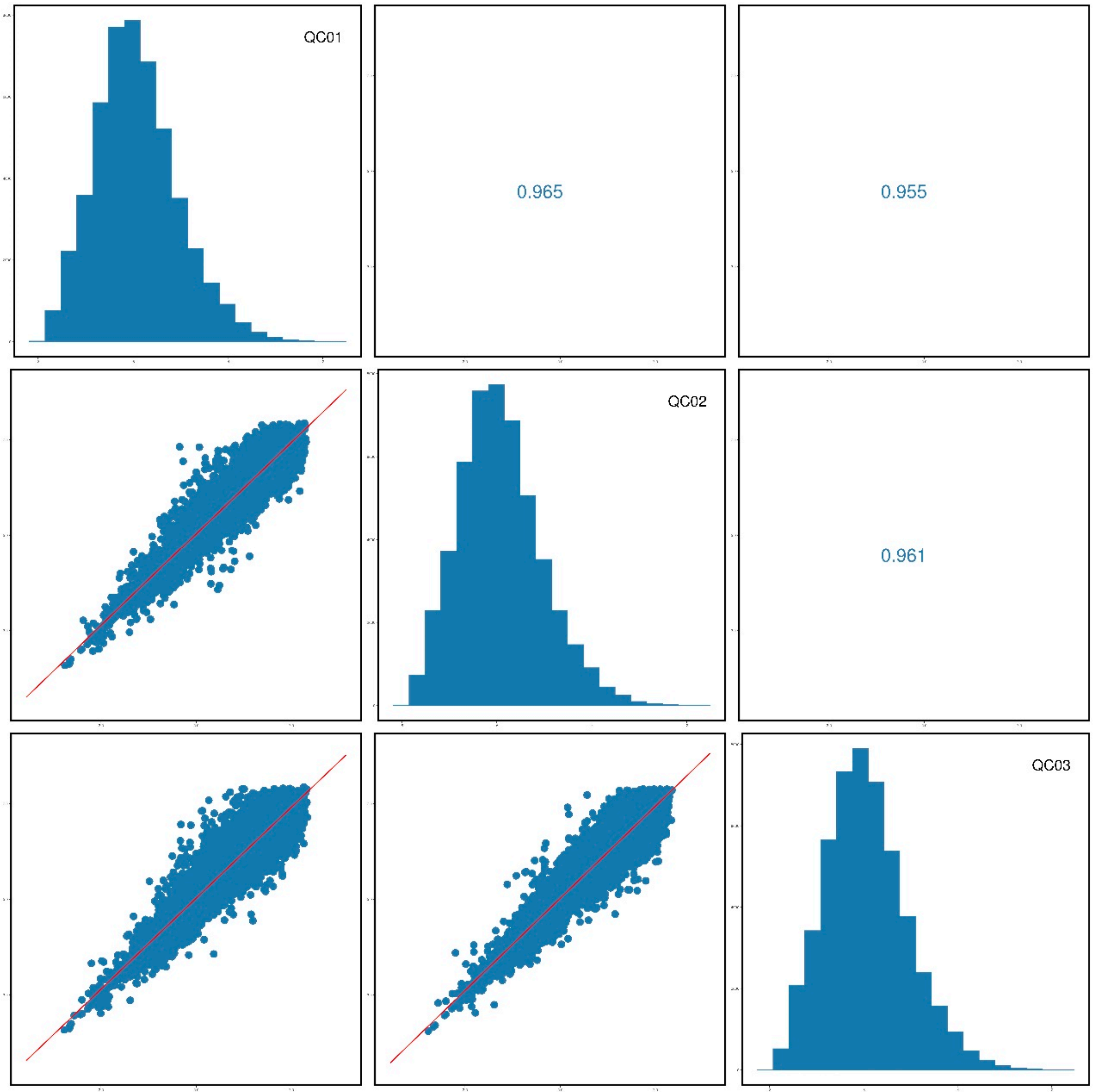

Figure S2. Pearson correlation coefficients between the three injections of QC sample based on the intensities of detected peaks obtained from the positive ionization mode.

Figure S3

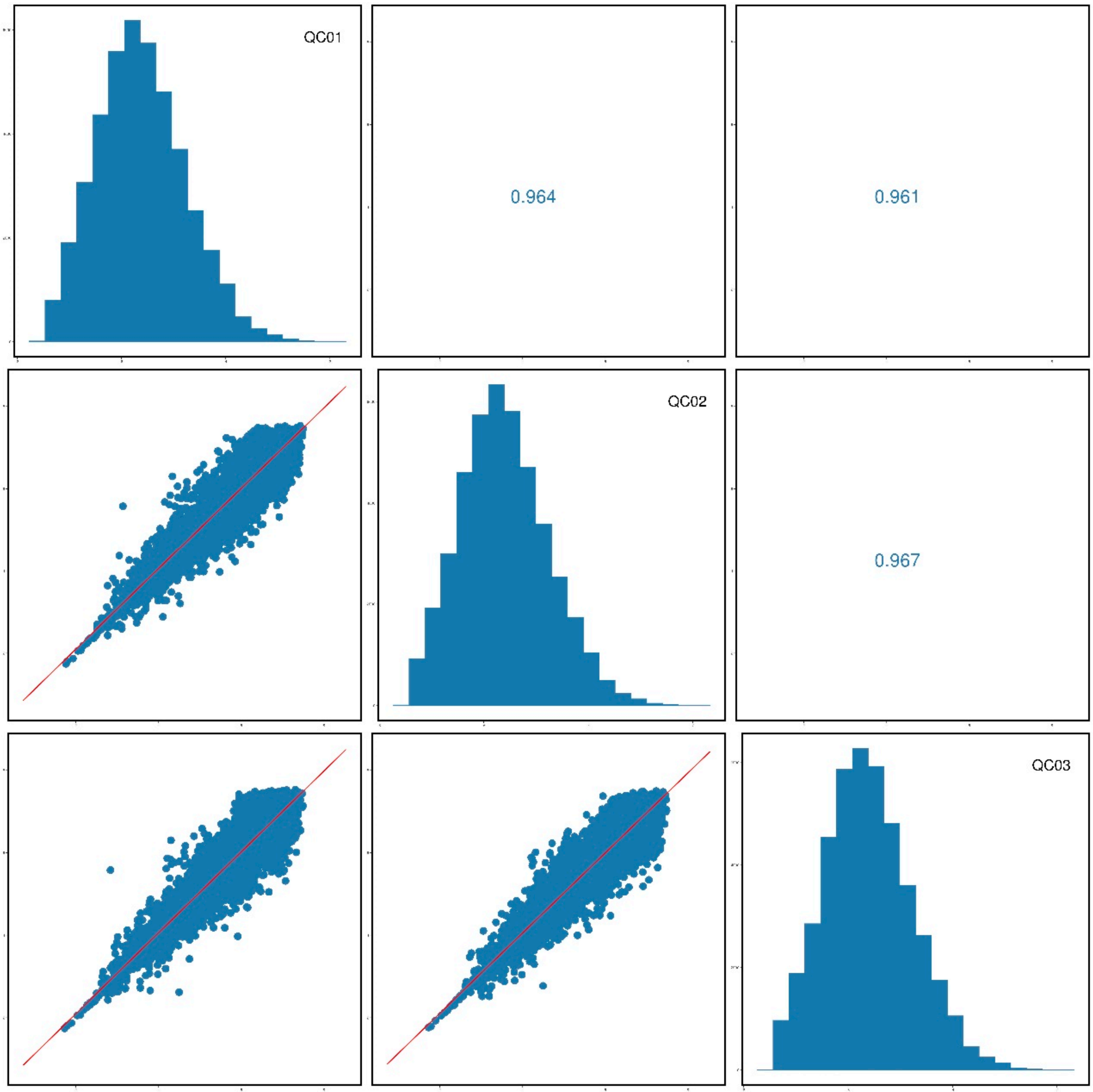

Figure S3. Pearson correlation coefficients between the three injections of QC sample based on the intensities of detected peaks obtained from the negative ionization mode.
